# Supplementary material for: Intergenerational transmission of the patterns of functional and structural brain networks
Source: iScience. 2021 Jun 11;24(7):102708. doi: 10.1016/j.isci.2021.102708 (PMC8253972; doi:10.1016/j.isci.2021.102708)
Supplement: Document S1. Figures S1–S7 [file mmc1.pdf]

iScience, Volume *24*

Supplemental information

Intergenerational transmission of the patterns of functional and structural brain networks

Yu Takagi, Naohiro Okada, Shuntaro Ando, Noriaki Yahata, Kentaro Morita, Daisuke Koshiyama, Shintaro Kawakami, Kingo Sawada, Shinsuke Koike, Kaori Endo, Syudo Yamasaki, Atsushi Nishida, Kiyoto Kasai, and Saori C Tanaka

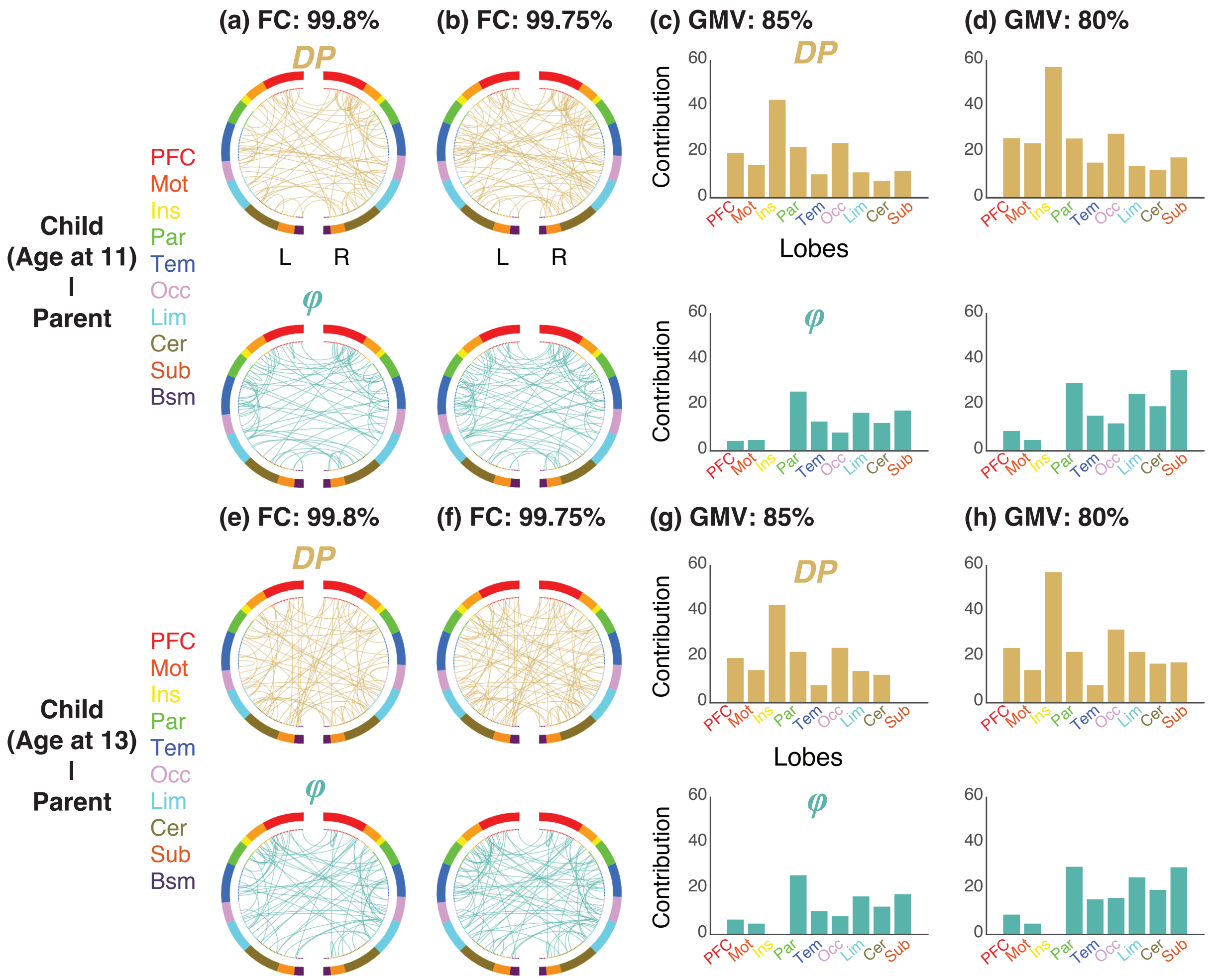

Figure S1: **Overall patterns of edges/regions with high differential power (highly discriminative among subjects) and group consistency (highly similar among subjects) tended to be similar across different thresholds and across development (Related to Figure 2)**

(a)–**(d)** child at age 11; **(e)**–**(h)** child at age 13. The figures show the results when the edges were thresholded at the 99.8th and 99.75th percentiles for FC, and regions were thresholded at the 85th, 80th percentiles for GMV. For each threshold, a circle plot (FC) and a bar graph (GMV) are shown, in which nodes are grouped according to anatomical location.

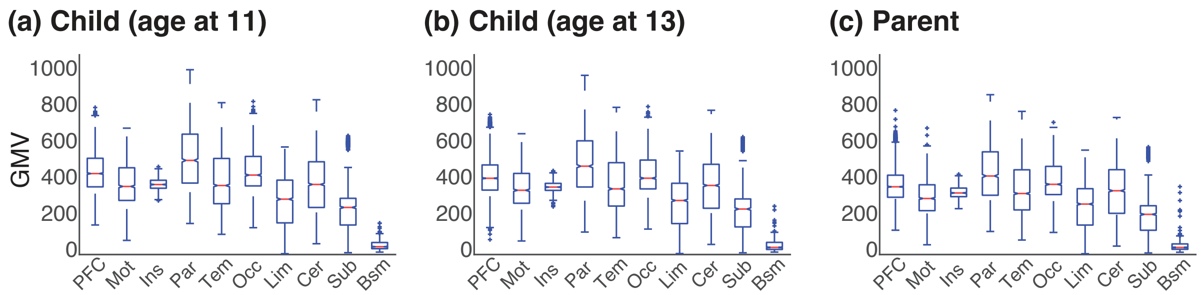

Figure S2: **Strengths of each lobule for GMV (Related to Figure 2)**

The red line indicates the median. The bottom and top edges of the box indicate the 25th and 75th percentiles, respectively. The crosses denote outliers, and the whiskers extend to the most extreme data points not considered outliers. The brainstem (the right-most boxes in each figure) exhibited much lower signal strength compared with the other lobules. Means ± s.e.m are shown.

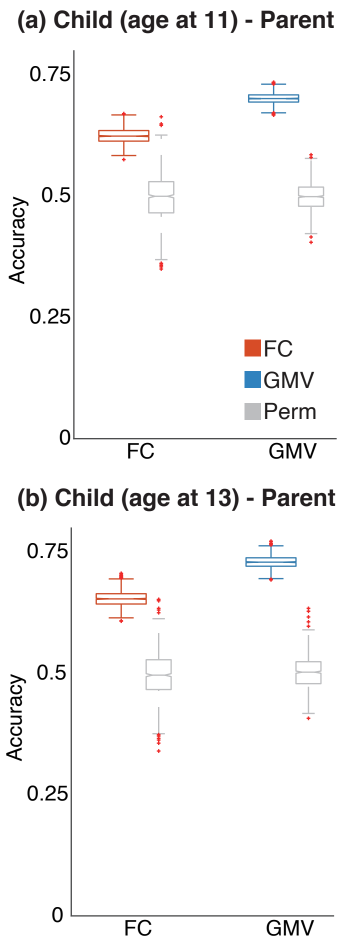

Figure S3: **Accuracies using only low-movement subjects (Related to Figure 2)**

We confirmed that qualitatively similar results were obtained when we excluded children whose head movements were in the top 25%, at both age 11 and age 13, resulting in the inclusion of 41.25% of the total sample.

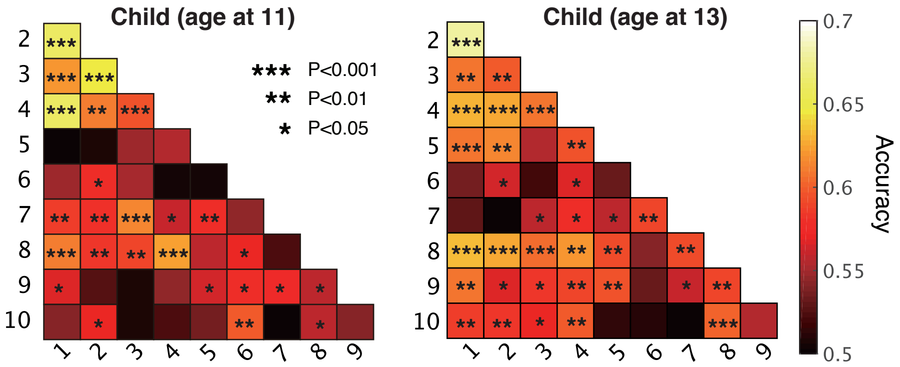

Figure S4: **Between-network analyses using only the between-network edges (Related to Figure 3)**

Statistical significance was assessed by comparing the distributions for each network obtained through bootstrapping, uncorrected. * 1,000 times permutation test, P < 0.05; ** P < 0.01; *** P < 0.001.

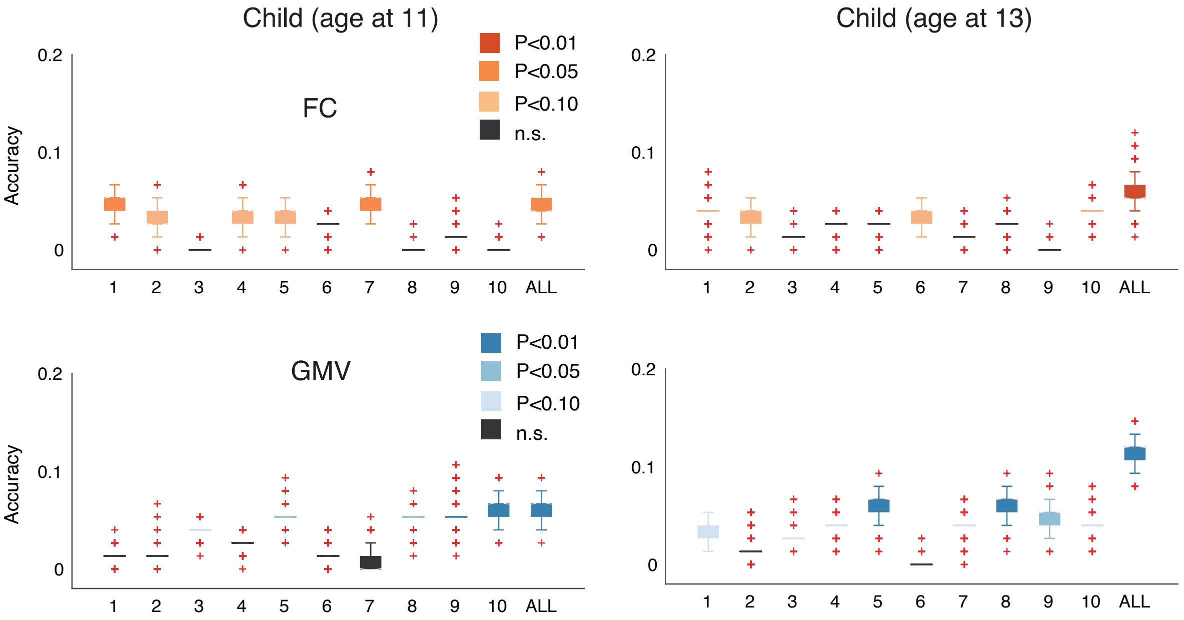

Figure S5: Accuracies obtained using conventional identification methods (Related to Figure 3)
Box plots of accuracies using FC (top row) and GMV (bottom row) for children at age 11 (left column) and 13 (right column) are shown. The bottom and top edges of the box indicate the 25th and 75th percentiles, respectively. The crosses denote outliers, and the whiskers extend to the most extreme data points not considered outliers. n.s. non-significant.

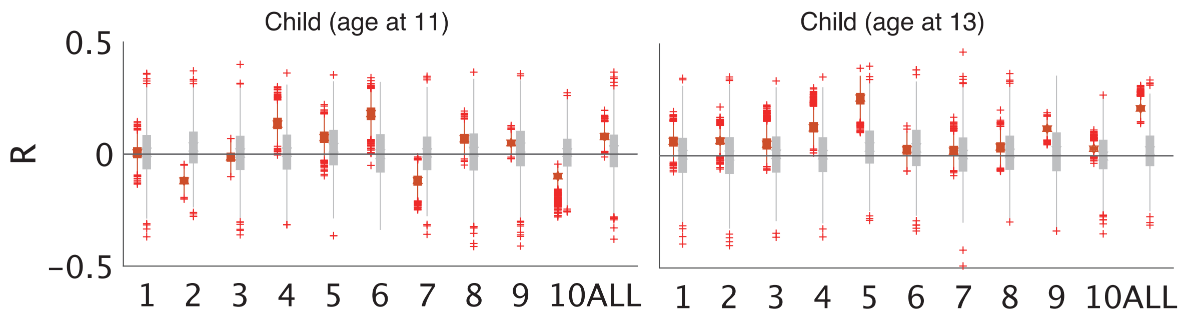

Figure S6 Pearson's correlation between the similarities defined by FC and GMV (Related to Table 2)

For each sub-network (1–10: sub-networks shown in Figure 3a) and whole-brain (ALL), we calculated Pearson's correlation coefficients between parent-child brain similarities defined by FC and those defined by GMV across all parent-child dyads (red box). We found that they exhibited relatively low correlations in general, suggesting that they contained independent information. Directly to the right of these red boxes (grey box) are the results of the 1,000-times permutation testing.

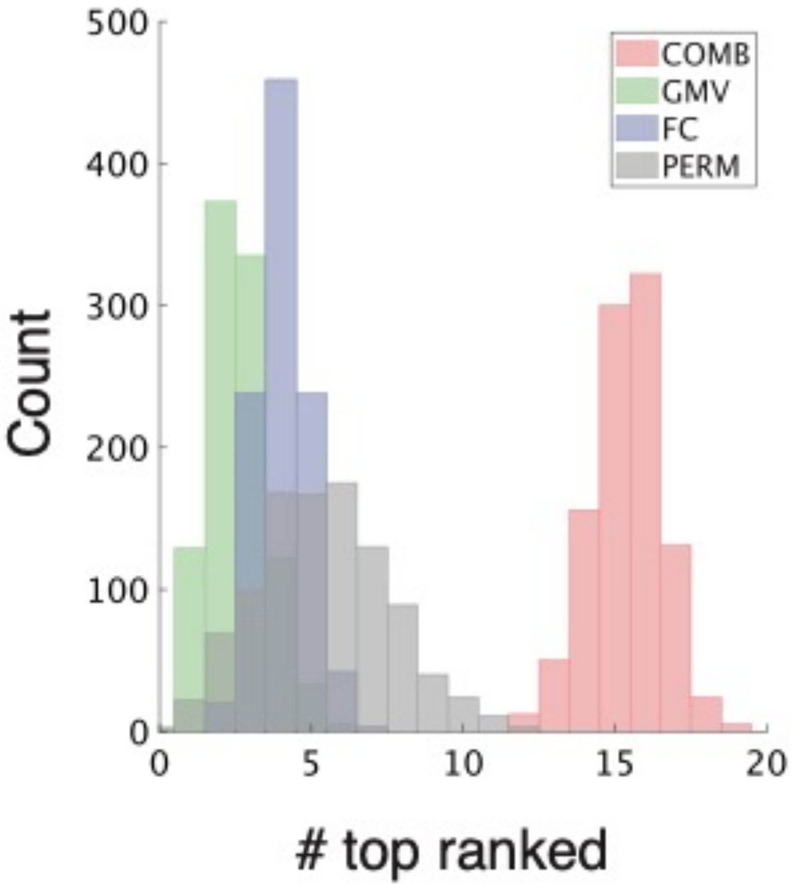

Figure S7: The number of top ranked networks for COMB, GMV and FC (Related to Table 2)

The number of top ranked networks for COMB (red bar), GMV (green bar), and FC (blue bar) estimated via bootstrapping and null distribution (grey bar) estimated via permutation are shown.
